# Legal Responsibility of the Drunkard

**Published:** 1909-06

**Authors:** 


**Legal Responsibility of the Drunkard.** By H. NORMAN BARNETT, F.R.C.S. London: Baillière, Tindall & Cox. 1908.

This excellent little book, prefaced with an introduction by Sir Andrew Reed, is an effort to supply a need which Mr. Barnett considers exists in the legal and medical professions with regard to the responsibility of the chronic drunkard, and pointing out in what respects the alcoholic condition renders a person responsible, and under what conditions he is not fit to exercise the legal rights of a sane person, as well as that the drunkard should not be treated as other criminals.

An introduction is followed by ten chapters which deal with "Crime and Responsibility," "Heredity," "Legal attitude towards and Legal Penalties for Drunkenness," &c. A most important chapter follows dealing with the "Legal Capacities of these Cases," and treats of their legal responsibilities, their ability to manage their own affairs, and to be accepted as witnesses. The last chapter is upon the "Recognition of the Disease." The book shows very clearly the real nature of the disease and the problem of inebriety, with the methods demanded in dealing with it.

The introduction by Sir Andrew Reed is a well put case for the medical treatment rather than punishment of such people. It seems strange nowaday why more criminal inebriates are not dealt with by committal to a reformatory, since the cost of these cases is borne by the State and not by the local authority. Medical men know, however, the great difficulties there are in getting inebriates committed to a reformatory, at all events in the present unsatisfactory state of the law, which gives them every chance of evading committal. Had numbers of such cases been treated in the early stage of the disease they might have been reformed, and have been spared ending their days in a lunatic asylum.

